# Manifestations of Salmonellosis in Small Ruminants in The Netherlands over a Period of 15 Years

**DOI:** 10.3390/ani16193031

**Published:** 2026-09-26

**Authors:** Nienke Snijders-van de Burgwal, Eveline Dijkstra, Annet Heuvelink, Erik van Engelen, Liesbeth Harkema, Piet Vellema, Carlijn ter Bogt-Kappert, René van den Brom

**Affiliations:** 1 Department of Small Ruminant Health, Royal GD, 7418 EZ Deventer, The Netherlands; e.dijkstra@gdanimalhealth.com (E.D.); l.harkema@gdanimalhealth.com (L.H.); p.vellema@gmail.com (P.V.); c.t.bogt@gdanimalhealth.com (C.t.B.-K.); r.vd.brom@gdanimalhealth.com (R.v.d.B.); 2 Department of Research and Development, Royal GD, 7418 EZ Deventer, The Netherlands; a.heuvelink@gdanimalhealth.com (A.H.); e.v.engelen@gdanimalhealth.com (E.v.E.)

**Keywords:** salmonellosis, small ruminants, sheep, goats, *Salmonella* Typhimurium, antimicrobial resistance, disease surveillance, outbreak investigation, one health

## Abstract

*Salmonella* spp. are major zoonotic pathogens and an important cause of foodborne disease. However, information on the occurrence of salmonellosis in small ruminants in The Netherlands is limited, and no specific control programme exists for sheep and goats. This study investigated temporal trends, serovar distribution, antimicrobial resistance patterns, and outbreak characteristics of salmonellosis in Dutch sheep and goats, using surveillance and diagnostic data from the national animal health monitoring system of Royal GD (GD). Overall, salmonellosis in small ruminants was relatively rare, but clear species-specific differences were observed among diagnosed cases, clinical presentation, serovar distribution, and epidemiological drivers. Between 2006 and 2020, *Salmonella* spp. were isolated from 34 sheep originating from 25 farms. No confirmed cases were recorded between 2006 and 2012 in goats; from 2013 onwards, 42 cases were diagnosed on 14 farms. In goats, the disease occurred predominantly during the neonatal period and was associated with environmental contamination, animal movements, limited therapeutic options, and, on some farms, also human cases of salmonellosis related to direct contact with the infected goat kids. Concurrent farm-level outbreaks caused by *Salmonella enterica* subsp. *enterica* serovar Typhimurium with shared or closely related MLVA profiles, together with human cases linked to infected dairy goat farms, underline the One Health relevance of salmonellosis in this sector. These findings highlight the need for species-specific approaches for surveillance, prevention, and control to support animal health management, prudent antimicrobial use, and mitigation of public health risks.

## Simple Summary

Salmonellosis is a bacterial disease that can affect sheep and goats and may also pose a risk to human health. We investigated cases of salmonellosis in sheep and goats in The Netherlands between 2006 and 2020 using data from the national animal health monitoring system. The disease was uncommon in sheep and was mainly associated with abortion, enteric disease, and septicaemia. From 2013 onwards, increased detection of salmonellosis in goats was recognised within the surveillance system and was associated with substantial herd-level disease. Disease mainly affected dairy goat kids and was characterised by diarrhoea, septicaemia, and increased mortality. Most outbreaks were caused by *Salmonella* Typhimurium and indications of persistence and spread within farms, with indications of transmission between farms and humans. These findings improve our understanding of salmonellosis in small ruminants and highlight its relevance to animal and public health.

## 1. Introduction

*Salmonella* spp. are important zoonotic pathogens and are a major cause of foodborne disease [1,2,3,4]. Infections occur in a wide range of animal species, and food products of animal origin constitute one of the main transmission routes to humans [2,5].

*Salmonella* spp. in small ruminants are reported worldwide, mainly in case and slaughterhouse reports, and can manifest in enteritis, septicaemia, and abortion, while subclinical infections also occur [3,6,7,8,9,10].

Information about salmonellosis in small ruminants in The Netherlands is limited. In contrast to national salmonella control programmes in poultry, pigs, and cattle [11,12,13], no specific programme exists for small ruminants and, consequently, government and industry lack an overview of its significance. Since 2013, *Salmonella enterica* subsp. *enterica* serovar Typhimurium (*S.* Typhimurium) infections have been reported by the Dutch animal health monitoring system for small ruminants as a cause of health problems on dairy goat farms [14,15]. These observations were the incentive for further investigation.

The aims of the present study were (I) to describe temporal patterns in *Salmonella* isolations in sheep and goat submissions for pathology, (II) to characterise isolated serovars and associated antimicrobial resistance patterns in both animal species, and (III) to describe the clinical course and outbreak dynamics on affected dairy goat farms.

## 2. Materials and Methods

### 2.1. Study Design and Case Definition

This retrospective observational study used data from the national monitoring and surveillance system for small ruminant health, coordinated by Royal GD (GD). This system has been active since 2002 and combines a veterinary consultancy helpdesk, information from farm visits, and diagnostic investigations, including voluntary post-mortem examinations submitted by farmers and veterinary practitioners [14]. Farmers and veterinary practitioners can be encouraged to do so if signals from other parts of the monitoring and surveillance system prompt such action. Approximately 1000 post-mortem examinations on sheep and goats are performed annually.

All small ruminant post-mortem examinations performed between 1 January 2006 and 31 December 2020 were screened in the GD Laboratory Information Management System (LIMS), and cases were retrieved through the GD data bank (GDDB). A case was classified as confirmed salmonellosis when compatible gross and/or histopathological lesions were present, and *Salmonella* spp. were isolated by bacterial culture.

### 2.2. Post-Mortem Examination and Data Collection

Post-mortem examinations at GD are performed by certified veterinary pathologists, according to standard operating procedures, and include gross pathological examination, with additional histopathology and diagnostics, such as bacteriology or toxicology, based on clinical history and macroscopic findings. In cases with a history of diarrhoea, tissue samples of both the small intestine and colon were collected for histology, and small intestinal contents were sampled for bacterial culture. In young lambs/kids, a lateral flow test of intestinal contents was included to test for *Cryptosporidium*, coronavirus, rotavirus, and *E. coli* K99. In cases involving abortion material, a dedicated sampling protocol is applied as described previously [16]. When clinical signs of the central nervous system were reported, a swab from the meninges and brain tissue was submitted for bacterial culture, combined with histologic examination of the brain. Furthermore, other lesional tissues were sampled for tailored additional testing based on the differential diagnosis for these lesions; this included histology, bacterial culture, PCR testing, and measurement of trace elements and heavy metal concentrations in the liver.

For each confirmed case, submission date, species, age, unique herd identification (UHI), and pathological diagnosis were extracted from pathology reports and submission forms. Age was primarily obtained from the national Identification and Registration (I&R) system of The Netherlands Enterprise Agency (RVO), based on the animal’s ear-tag identification number [17]. When no ear tag was available or the number could not be verified, the age reported in the clinical history on the submission form was used. Clinical information provided by the farmer or their veterinary practitioner was reviewed to document reported morbidity, mortality, and disease course at flock or herd level.

### 2.3. Laboratory Analysis

Samples collected during post-mortem examination were cultured using routine diagnostic procedures. Tissue samples were inoculated onto sheep blood agar plates and selective xylose lysine deoxycholate (XLD) agar plates. To improve detection of *Salmonella* spp., samples were additionally enriched in buffered peptone water followed by selective enrichment in modified semi-solid Rappaport–Vassiliadis (MSRV) medium. Suspected colonies were subcultured on XLD agar and brilliant green agar with novobiocin and finally identified by matrix-assisted laser desorption ionization time-of-flight mass spectrometry (MALDI-TOF MS; MALDI Biotyper, Bruker Daltonics GmbH & Co. KG, Bremen, Germany).

Confirmed *Salmonella* isolates were serotyped by agglutination of somatic (O) and flagellar (H) antigens according to the Kauffmann–White–Le Minor scheme at GD and/or the National Institute for Public Health and the Environment (RIVM). Isolates from goat post-mortem submissions obtained in 2016 were additionally submitted to RIVM for multiple-locus variable-number tandem-repeat analysis (MLVA), a molecular typing technique that characterises bacterial isolates based on variation in repetitive DNA sequences.

Antimicrobial susceptibility testing (AST) was performed at GD. Until mid-2013, susceptibility was assessed by agar diffusion using Neo-Sensitabs (Rosco Diagnostica A/S, Taastrup, Denmark). The routine panel included ampicillin, gentamicin, neomycin, spectinomycin, colistin, trimethoprim-sulfamethoxazole, and flumequine. Isolates tested against flumequine were additionally evaluated for enrofloxacin susceptibility. From mid-2013 onwards, AST was performed by broth microdilution using the MICRONAUT system (Merlin Diagnostics GmbH, Bornheim, Germany). Minimum inhibitory concentrations (MICs) were determined according to the Clinical and Laboratory Standards Institute (CLSI) guidelines applicable at the time of testing [18]. Customised microtiter plates routinely used at the GD bacteriology laboratory contained serial two-fold dilutions of antimicrobial agents representing the aminoglycoside, cephalosporin, quinolone, phenicol, macrolide, penicillin, polymyxin, tetracycline, pleuromutilin, and trimethoprim/sulfonamide classes. MIC values were read photometrically and interpreted using Merlin Advanced Expert System software (MCN-6) [19]. Isolates were classified as susceptible, intermediate, or resistant using CLSI veterinary breakpoints where available; for clindamycin and trimethoprim-sulfamethoxazole, human-derived breakpoints were applied [18]. Both the agar diffusion and broth microdilution methods have been extensively validated before introduction in the routine laboratory. To ensure the quality of susceptibility testing results, on each testing day, the standard quality control strain *E. coli* ATCC 25922 was included with known inhibition zone diameters and MIC values. The change in AST methodology during the study period, differences between antimicrobial panels, and the fact that some antimicrobials were tested only in subsets of isolates limit direct comparison of resistance proportions across the entire study period. Antimicrobials were classified according to the European Medicines Agency (EMA) categorisation into four groups: A (Avoid), B (Restrict), C (Caution) and D (Prudence) [EMA/AMEG]. According to EMA, AMEG category B antimicrobials are considered critically important for human medicine and should be used in animals only when no appropriate alternatives are available. Each antibiotic tested was assigned to one EMA category, and this variable was included in the analysis of resistance patterns [20]. Multidrug resistance (MDR) was defined as resistance to at least one antimicrobial agent in three or more antimicrobial categories [21].

### 2.4. Analysis of Retrospective Surveillance Data

Data from all confirmed cases identified through post-mortem surveillance between 2006 and 2020 were analysed descriptively. Cases were summarised by year, species, and age category to assess temporal patterns in *Salmonella* detections. Serovar distributions were summarised for all available isolates, and AST results were described by isolate and antimicrobial agent.

### 2.5. Outbreaks on Dairy Goat Farms

To further characterise outbreak dynamics at farm level, all cases of salmonellosis in goats between 2016 and 2020 involving two or more animals on the same farm were selected from the LIMS database. Following pathological and bacteriological examination, farmers and their attending veterinarians were contacted to obtain additional information on the outbreaks. As part of the standard procedures of the Dutch small ruminant health surveillance programme, farm visits are planned in consultation with the animal owner and the attending veterinarian. Participation in farm visits and follow-up investigations is voluntary. Additional epidemiological information regarding herd management, animal health, biosecurity, antimicrobial use, and outbreak history was collected only after agreement of the participating farmer and veterinarian. Five farms were subsequently visited by a small ruminant specialist, registered as such by the European College of Small Ruminant Health Management. Two farms with consecutive annual confirmations of salmonellosis were visited on three additional occasions, during which samples were collected. During these visits, additional information was collected regarding herd size, kidding pattern, biosecurity, antimicrobial use, clinical presentation, and estimated morbidity and mortality. Environmental samples were collected for bacteriological examination. Boot swabs were used to sample bedding and floor surfaces in rearing areas and pens, and pen partitions were sampled using sterile washcloths [22,23]. Bulk tank milk samples were also collected for bacteriological culture. Isolates were further characterised by serotyping and MLVA as described above. Collected information was evaluated descriptively, with the aim of characterising clinical presentation and within-farm outbreak dynamics.

## 3. Results

### 3.1. Temporal Patterns and Case Characteristics

Between 2006 and 2020, 11,402 small ruminant post-mortem examinations were recorded in the GD LIMS database, of which 4230 involved goats and 7172 involved sheep, and 76 confirmed cases of salmonellosis were identified.

In sheep, *Salmonella* spp. were isolated from 34 individual animals originating from 25 farms. In goats, 42 cases were diagnosed, originating from 14 farms. No cases were recorded in goats between 2006 and 2012. The numbers peaked in 2017 (*n* = 12; five farms) (Figure 1).

### 3.2. Serovars and Antimicrobial Resistance Patterns

#### 3.2.1. Sheep

The case characteristics, pathological diagnoses, and serotypes of sheep cases are shown in Table 1. Sixteen out of 34 cases concerned aborted lambs. Clinical symptoms in the remaining submissions included flock-level disease with (sudden) deaths, anorexia, diarrhoea, dullness, and acute mortality in weaned lambs shortly after weaning. Production losses, notably reduced growth and diarrhoea, were highlighted as major features in relatively few submissions.

Salmonellosis affected sheep of various ages and multiple organ systems. In addition to cases from abortion submissions, intestinal disorders (13/34), sepsis (4/34), and laryngitis (1/34) were found.

*Salmonella enterica* subsp. *enterica* serovar Typhimurium (*S.* Typhimurium; *n* = 3), *S.* Dublin (*n* = 11) and *Salmonella enterica* subsp. *diarizonae* (*S. enterica* subs. *diarizonae; n* = 3) were identified in 3, 11, and 3 out of 17 cases, respectively. The remaining 17 isolates could not be serotyped.

Antimicrobial susceptibility testing (AST) results are summarised in Table 2 and were available for 31 out of 34 sheep isolates (agar diffusion, *n* = 17; broth microdilution, *n* = 14). Briefly, resistance was most consistently observed to tiamulin, tilmicosin, and tylosin, to which *Salmonella* spp. are known to exhibit intrinsic resistance, and resistance was frequently found to sulfonamides. In contrast, susceptibility was retained for several commonly tested antimicrobials (e.g., ampicillin, colistin, gentamicin, neomycin, trimethoprim–sulfonamides). Importantly, no resistance was detected to AMEG category B (‘Restrict’) antimicrobials, including fluoroquinolones and third-/fourth-generation cephalosporins, nor to several other commonly tested antimicrobials such as ampicillin, gentamicin, neomycin, tetracycline, and trimethoprim–sulfonamides, and no multidrug-resistant isolates were identified.

#### 3.2.2. Goats

The case characteristics, pathological diagnoses and serotypes of the 42 cases in goats are shown in Table 3. In contrast to the heterogeneous presentation observed in sheep, cases in goats were dominated by neonatal disease: forty out of 42 cases involved kids, whereas only two concerned adult animals (>1 year). In 34 cases, farmers and/or veterinary practitioners reported acute morbidity and mortality of multiple kids, with or without diarrhoea. Neurological and respiratory symptoms were mentioned incidentally. On one of the farms with kid mortality, a decreased milk production was observed by the farmer.

Despite the predominance of disease in young kids, *Salmonella* spp. were only once identified in goat abortion submissions. The main pathological features in the submitted kids were intestinal disorders with or without septicaemia (40/42).

Serotyping revealed a predominance of *S.* Typhimurium (*n* = 35), one *S.* Dublin isolate, one isolate of *S.* Enteritidis, and five isolates in serogroup B (not further serotyped).

Antimicrobial susceptibility testing (AST) was performed for all isolates (agar diffusion, *n* = 4; broth microdilution, *n* = 38). High levels of resistance were observed to ampicillin, sulfonamides, tetracycline and trimethoprim-containing compounds, whereas isolates also showed limited susceptibility to tiamulin, tilmicosin and tylosin, antimicrobials for which intrinsic reduced susceptibility in *Salmonella* should be considered when interpreting AST results (Table 4). No resistance was detected to AMEG category B antimicrobials, including fluoroquinolones and third-/fourth-generation cephalosporins. Multidrug resistance was identified in 35 isolates.

The single *S.* Dublin goat isolate showed intermediate susceptibility to colistin, florfenicol, spectinomycin and streptomycin, and resistance to sulfonamides, tiamulin, tilmicosin and tylosin. The *S.* Enteritidis isolate showed intermediate susceptibility to florfenicol, and resistance to tiamulin, tilmicosin, and tylosin.

### 3.3. Clinical Course and Outbreak Dynamics in Goats

Between 2016 and 2020, salmonellosis outbreaks were confirmed on 11 dairy goat farms after post-mortem examination. Five goat farms, one buck-fattening and four dairy farms, were visited for on-site investigation and additional sampling (Table 5). No other livestock species were present on these farms. All outbreaks involved *S.* Typhimurium in goat kids aged one to three weeks, causing diarrhoea, reduced milk intake, lethargy, and mortality. According to the farmers and their veterinary practitioners, antibiotic treatments (e.g., trimethoprim–sulfonamide, neomycin, paromomycin, colistin) were ineffective. In addition to antibiotic treatments, four farmers completely depopulated, thoroughly cleaned and disinfected their rearing facilities aiming at a long-term solution. On two farms, no new cases of salmonellosis were detected after implementation of these measures. In contrast, recurrence on the remaining two farms suggested that control measures were not fully effective. One farmer administered an autovaccine to all his pregnant goats during late pregnancy for two consecutive years, aiming at producing colostral antibodies to *Salmonella* spp., and reported a perceived positive effect, although clinical infections in goat kids could not completely be prevented. Environmental sampling resulted in the detection of *S.* Typhimurium in two dairy pens and in most kid-rearing areas, even on those farms where earlier depopulated facilities had been cleaned and disinfected. Bacterial culture of all bulk tank milk samples was negative for *Salmonella* spp.

Animal movements between farms appeared to be a plausible explanation for introduction in some cases, as outbreaks occurred on three farms after animal movements from infected farms. Additionally, goat kids on two farms received bovine colostrum originating from dairy cattle herds with an unknown salmonella status.

Human illness concurrent with goat salmonellosis outbreaks was reported on three farms. On one farm, *S.* Typhimurium isolates with identical MLVA profiles were detected in three children with gastrointestinal disease and affected goats. On a second farm, a family member who worked on the farm became severely ill and was hospitalised, requiring admission to an intensive care unit. The same *S.* Typhimurium MLVA profile was identified in both blood samples obtained from this patient and animal samples. This MLVA profile was rare in The Netherlands and had previously been detected only three times, in 2014 and 2015. On a third farm, a person working on the farm was reported to have chronic gastrointestinal complaints. No additional diagnostic investigations were conducted to establish the underlying aetiology of these complaints.

## 4. Discussion

*Salmonella* spp. are major zoonotic pathogens which have been isolated from a wide range of animal species, and for which national control programmes exist in The Netherlands for poultry, pigs, and cattle [11,12,13]. For sheep and goats, national control programmes for salmonellosis are not available as there has been no incentive for such programmes. Severe outbreaks of salmonellosis with high morbidity and mortality in kids on dairy goat farms prompted this retrospective study. These outbreaks have been reported since 2016, and were mainly caused by *S.* Typhimurium, the source of which is unknown.

Between 2006 and 2020, *Salmonella* spp. were isolated from less than one per cent of 11,402 small ruminant submissions for post-mortem examination. The isolated *Salmonella* spp. were particularly associated with abortion, enteric disease, septicaemia, and, in goats, severe herd-level outbreaks in young kids. In sheep, *Salmonella* spp. were isolated throughout the study period. In goats, no cases were identified between 2006 and 2013. The occurrence of severe outbreaks in kids on dairy goat farms since 2016 may indicate a true change in disease occurrence; however, the available data do not allow to distinguish between an epidemiological change and increased detection as a consequence of submission behaviour, diagnostic intensity, or surveillance sensitivity. This limitation is inherent to a monitoring system based on a combination of a veterinary consultancy helpdesk, information from farm visits, and voluntary microbiological diagnostics and post-mortem examinations, particularly when numbers of submitted animals do not necessarily reflect the magnitude of disease at flock or herd level [14]. However, submissions can be encouraged if signals from other parts of the monitoring and surveillance system prompt such action. Although a systematic population-level surveillance does not exist, our findings nonetheless suggest a rise in salmonellosis in dairy goat farms since 2016.

Clear differences were observed between sheep and goats. In sheep, almost half of the cases concerned abortion submissions with *S.* Dublin as the main cause. *Salmonella* spp. typically associated with abortion in sheep, like *S.* Abortusovis, *S.* Indiana, *S.* Montevideo, and S. Brandenburg, were not found. Notably, *S.* Dublin is also recognised as a cause of abortion in sheep elsewhere [25,26,27]. In addition, *S. enterica* subsp. *diarizonae* was identified in association with abortion, enteric disease, and septicaemia; however, its role is unclear as *S. enterica* subsp. *diarizonae* is regularly isolated from healthy sheep [28,29]. The associated clinical presentations and pathological findings in the present study support a potential pathogenic role. In the remaining cases, intestinal disorders and septicaemia, caused by *S.* Typhimurium and *S.* Dublin, were the most important.

In goats, with a few exceptions, salmonellosis occurred only on dairy goat farms or at dairy kid rearing facilities as a severe herd-level outbreak of intestinal disorders with or without septicaemia in young kids and was mainly caused by *S.* Typhimurium. Experimental infections in goats have also shown that *S.* Typhimurium primarily induces gastrointestinal disease [30]. Both observations are in line with studies from other countries where *S.* Typhimurium and *S.* Enteritidis are most frequently found in caprine faeces or organs [3,7,31,32]. The serovar distribution in our study is therefore unlikely to be incidental and may reflect a combination of host species differences and serovar-specific factors, including age at exposure, transmission opportunities, and host ecology [9,28,33].

Farm visits confirmed the above-mentioned observations. Affected kids were between one and three weeks of age and presented with diarrhoea, reduced milk intake, lethargy, and mortality. Although relatively few kids per farm were submitted for post-mortem examination, these submissions often represented large herd-level outbreaks with substantial morbidity and mortality. Similar patterns have been described in dairy calves, where the rearing environment functions as an amplification point for transmission [34,35]. No *Salmonella* spp. were isolated from bulk tank milk samples, and only incidentally found in environmental samples collected in the facilities of adult goats, suggesting that infection was concentrated in rearing units, although ongoing circulation elsewhere on the farm and intermittent shedding by adult carriers cannot be excluded. Together, these observations may indicate that disease transmission primarily occurred within kid-rearing units, although formal risk factor studies are required to identify determinants of transmission.

The recurrence of salmonellosis despite depopulation and thorough cleaning and disinfection possibly indicates environmental persistence or intermittent shedding by adult carriers, although re-introduction cannot be excluded. However, recurring detection of comparable MLVA profiles in subsequent years makes repeated introduction less likely. Persistence has been described in cattle and pig production systems, where *Salmonella* spp. may survive in housing, slurry, and contaminated surfaces, and can be re-isolated months after control measures have been implemented [36,37,38,39]. These observations highlight that the performance of cleaning and disinfection should be regarded as a critical control point requiring validation under field conditions. Future studies should therefore investigate the effectiveness of different cleaning and disinfection strategies, the duration of environmental persistence, optimal sampling sites for surveillance, and whether the phenomenon of ‘persistent carriers’ also occurs in goats.

Antimicrobial therapy was perceived as having limited impact on outbreak control despite the use of multiple antimicrobial classes, a finding that mirrors observations from multidrug-resistant *S.* Typhimurium outbreaks in dairy calves [35,40]. Effects of management interventions such as vacancy periods and enhanced hygiene measures were variable and sometimes temporary. One farmer reported a perceived effect of an autogenous vaccine, but evidence for efficacy is limited. In swine, autogenous inactivated *S.* Typhimurium vaccines have been associated with reduced shedding [41], whereas an autogenous *S.* Typhimurium and *S.* Dublin vaccine in calves induced high antibody titres without demonstrated effects on excretion or clinical outcomes [42].

Retrospective observational studies inevitably encounter changes in methodology over time that complicate the interpretation of results. In our study, AST methodology changed from agar diffusion to broth microdilution, antimicrobial panels were not identical, and not all isolates were tested extensively. In addition, comparisons of serovar distributions and antimicrobial susceptibility patterns across years should be interpreted with caution because serotyping was not available for all isolates, antimicrobial susceptibility testing methodology changed during the study period, and the number of isolates per year was low (generally <20 per species per year). Therefore, temporal trend analyses of serotypes and antimicrobial resistance patterns were not performed. We found that antimicrobial susceptibility patterns differed markedly between sheep and goats, except for tiamulin, tilmicosin, and tylosin to which resistance was consistently observed in both species. The latter observation should be interpreted with caution because of the intrinsic poor susceptibility of *Salmonella* spp. to pleuromutilins and macrolides [24]. Acquired resistance was generally uncommon among sheep isolates, whereas goat isolates frequently showed resistance to ampicillin, sulfonamides, tetracycline, trimethoprim-containing compounds, and several aminoglycosides. No resistance was detected to AMEG category B antimicrobials, including fluoroquinolones and third-/fourth-generation cephalosporins, which is encouraging from both veterinary and public health perspectives [20]. According to the established multidrug resistance (MDR) definition, namely resistance to at least one antimicrobial agent in three or more antimicrobial classes, a considerable proportion of goat isolates exhibited multidrug-resistant phenotypes, which may have contributed to the limited therapeutic response reported during outbreaks [21]. The perceived lack of treatment efficacy possibly also reflects the importance of environmental contamination, high infection pressure, and rapid transmission among young animals, emphasising that outbreak control depends primarily on biosecurity and management measures rather than antimicrobial treatment alone.

Available farm-level data were compatible with the introduction and dissemination of *Salmonella* spp. through animal movements between farms. Shared suppliers, reported animal movements, and the presence of multiple linked locations under single ownership together suggest epidemiological connections through which transmission between farms, or between facilities within the same operation, may have occurred. The similarity of MLVA profiles among isolates from linked farms was likewise suggestive of relatedness between at least some isolates. However, given the observational nature of the available data and the limited discriminatory resolution of MLVA compared with whole-genome sequencing, these findings cannot distinguish between direct transmission, environmental persistence, or repeated introduction from a common source. Therefore, the present data identify epidemiological links that are compatible with dissemination of *S.* Typhimurium through animal movements. These observations are consistent with wider evidence that inter-farm connectivity may contribute to spread of *Salmonella* spp. within livestock systems and may facilitate the circulation of successful or resistant clones across production networks [7,40].

The zoonotic implications of these outbreaks warrant particular emphasis. Human illness was reported on three affected farms, ranging from self-limiting gastrointestinal disease to severe systemic illness. Identical *S.* Typhimurium MLVA profiles were identified in isolates from humans and goat kids, supporting a close epidemiological link. Notably, this MLVA profile was rare in The Netherlands and had been detected only three times previously, in 2014 and 2015. Although these observations do not allow conclusions regarding the direction of transmission, the occurrence of identical and uncommon MLVA profiles in both humans and goats strengthens the epidemiological linkage. Although the negative bulk tank milk samples are reassuring from a food-safety perspective, they do not eliminate the possibility of transmission through other routes, including direct contact with infected kids, exposure to contaminated housing environments, or household contact on family-run farms. Such pathways are especially relevant in dairy goat systems, where close and repeated contact between animals and humans is common. Similar clusters involving closely related *S.* Typhimurium strains in animals and humans have been described on farms, emphasising that salmonellosis is a One Health problem affecting both animal and human health [7,43,44,45]. These findings argue for integrated outbreak investigations in which animal, environmental, and human data are evaluated together to improve farm-level control and to identify exposure pathways that might otherwise remain unrecognised.

Retrospective observational studies inevitably encounter limitations, as mentioned above. Furthermore, detailed farm-level information regarding herd size, management practices, biosecurity measures, treatment protocols, and production records was not consistently available for all investigated farms because data collection relied on voluntary participation and retrospective surveillance records. Consequently, the level of detail available differed between farms, and not all potentially relevant epidemiological information could be evaluated. In addition, it is difficult to determine at what stage the wider epidemiological relevance of early cases could have been recognised. Results of multiple submissions from one dairy goat farm in 2015 were initially interpreted as a herd-specific problem until further outbreaks from 2016 onwards became apparent. Nevertheless, this study provides a valuable long-term overview of salmonellosis in Dutch sheep and goats and highlights the clinical and epidemiological significance of the disease in both species.

## 5. Conclusions

Between 2006 and 2020, salmonellosis was detected in less than one per cent of 11,402 small ruminant submissions for post-mortem examination, but clear differences were observed between sheep and goats. In sheep, infections occurred sporadically at various ages and were mainly associated with abortion, enteric disease, and septicaemia, with *S.* Dublin being the predominant serovar. In goats, several cases have been confirmed since 2016, and these cases were frequently associated with herd-level disease, particularly in young kids, and most often linked to *S.* Typhimurium. The occurrence of recurrent outbreaks, antimicrobial resistance to several commonly used antimicrobials, and epidemiological links between farms highlight the need for species-specific continued surveillance and prevention and control strategies for the Dutch small ruminant population.

## Figures and Tables

**Figure 1:**
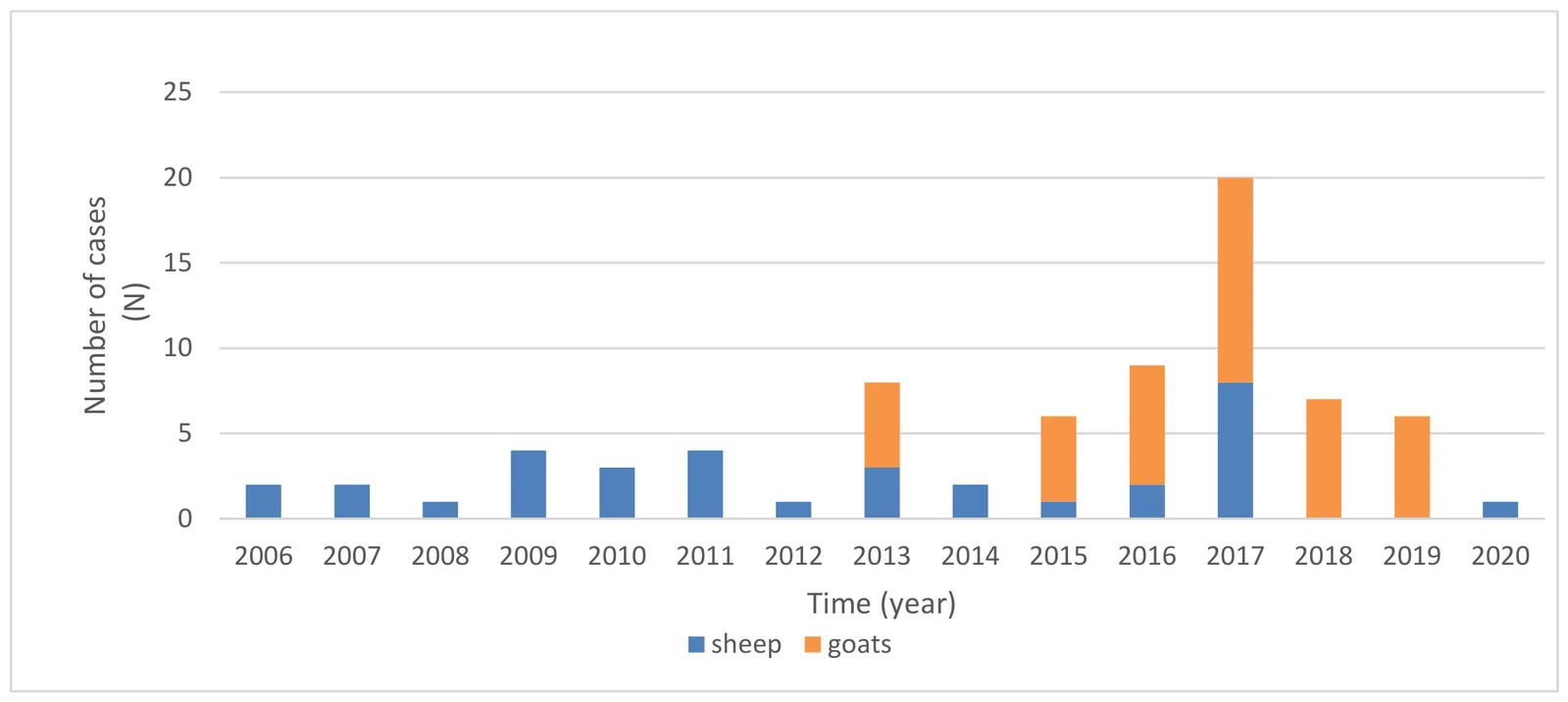
Annual numbers of confirmed cases of salmonellosis identified through post-mortem examination.

**Table 1:** Case characteristics, pathological diagnoses, and serotypes of 34 sheep salmonellosis cases.

Age Indication (Days) **	Year	Month	Type of Presentation *	Pathological Presentation	*Salmonella* Serotype or Serogroup
0	2006	December	Unknown	Abortion	*S.* Dublin
0	2006	December	Unknown	Abortion	*S.* Dublin
0	2009	April	Individual	Abortion	Unknown
0	2009	September	Individual	Abortion	*S.* Dublin
0	2009	September	Individual	Abortion	*S.* Dublin
0	2011	January	Outbreak	Abortion	Unknown
0	2011	January	Outbreak	Abortion	Unknown
0	2011	March	Individual	Abortion	Unknown
0	2014	February	Outbreak	Abortion	Unknown
0	2017	January	Individual	Abortion	*S.* Dublin
0	2017	August	Outbreak	Abortion	*S.* Dublin
0	2017	August	Outbreak	Abortion	*S.* Dublin
0	2017	August	Outbreak	Abortion	*S.* Dublin
0	2017	August	Outbreak	Abortion	*S.* Dublin
0	2017	August	Outbreak	Abortion	*S.* Dublin
0	2020	December	Outbreak	Abortion	*S. enterica* subsp. *diarizonae*
1	2009	May	Individual	Septicaemia	Unknown
3	2020	September	Individual	Septicaemia	*S. enterica* subsp. *diarizonae*
21	2007	February	Individual	Septicaemia	*S.* Typhimurium
42	2014	December	Outbreak	Intestinal disorder	Unknown
233	2012	November	Outbreak	Intestinal disorder	*S.* Typhimurium
244	2013	September	Outbreak	Intestinal disorder	Unknown
365	2010	March	Outbreak	Intestinal disorder	Unknown
596	2011	November	Outbreak	Intestinal disorder	Unknown
725	2013	March	Outbreak	Intestinal disorder	Unknown
1210	2010	March	Outbreak	Intestinal disorder	Unknown
1222	2016	August	Outbreak	Intestinal disorder	*S. enterica* subsp. *diarizonae*
1392	2017	January	Outbreak	Laryngitis	Unknown
1447	2016	June	Outbreak	Intestinal disorder	Unknown
no data	2007	March	Unknown	Septicaemia	*S.* Dublin
no data	2008	January	Unknown	Intestinal disorder	Unknown
no data	2010	October	Unknown	Intestinal disorder	*S.* Typhimurium
no data	2013	April	Unknown	Intestinal disorder	Unknown
no data	2015	March	Outbreak	Intestinal disorder	Unknown

* Outbreak is defined as more than one animal with comparable clinical features at submission; ** ‘Age’ indicates the age of the submitted animal at death or abortion.

**Table 2:** Antimicrobial susceptibility test results for 31 out of 34 isolates of *Salmonella* spp. from sheep.

Antibiotics	AMEG Category ª	Number of Samples	Number (%) of Intermediate Isolates	Number (%) of Resistant Isolates
Amoxicillin–clavulanic acid	D	14	0 (0)	0 (0)
Ampicillin	D	31	0 (0)	0 (0)
Apramycin	C	14	0 (0)	0 (0)
4th generation cephalosporins	B	14	0 (0)	0 (0)
Cefotaxime	B	14	0 (0)	0 (0)
Colistin	B	31	6 (19)	0 (0)
Enrofloxacin	B	14	0 (0)	0 (0)
Florfenicol	C	14	5 (36)	0 (0)
Flumequine	B	31	0 (0)	0 (0)
Gentamicin	C	31	0 (0)	0 (0)
Neomycin	C	31	0 (0)	0 (0)
Spectinomycin	D	31	15 (48)	0 (0)
Sulfonamides	D	14	0 (0)	11 (79)
Tetracycline	D	14	0 (0)	0 (0)
Tiamulin *	C	14	0 (0)	14 (100)
Tilmicosin *	C	14	0 (0)	14 (100)
Streptomycin	C	14	1 (7)	0 (0)
Trimethoprim	D	14	0 (0)	0 (0)
Trimethoprim−sulfonamides	D	31	0 (0)	0 (0)
Tylosin *	C	14	0 (0)	14 (100)

ª EMA’s Antimicrobial Advice Ad Hoc Expert Group (AMEG) categorisation: B = Restrict; C = Caution; D = Prudent [20]. * The organism is intrinsically resistant to the indicated antimicrobial agent(s) [24].

**Table 3:** Case characteristics, pathological diagnoses, and serotypes of 42 goat salmonellosis cases.

Age Indication (Days) **	Year	Month	Type of Presentation *	Pathological Presentation	*Salmonella* Serotype or Serogroup
0	2013	January	Outbreak #	Abortion	*S.* Typhimurium
3	2016	May	Outbreak	Intestinal disorder	*S.* Typhimurium
4	2019	May	Outbreak	Intestinal disorder	*S.* Typhimurium
5	2016	March	Outbreak	Intestinal disorder	*S.* Typhimurium
5	2017	April	Outbreak ##	Intestinal disorder	*S.* Typhimurium
5	2018	March	Outbreak	Intestinal disorder	*S.* Typhimurium
5	2018	March	Outbreak	Intestinal disorder	*S.* Typhimurium
5	2019	May	Outbreak	Intestinal disorder	*S.* Typhimurium
6	2016	May	Outbreak	Intestinal disorder	*S.* Typhimurium
6	2017	March	Outbreak	Intestinal disorder	*Salmonella* serogroup B
6	2017	July	Outbreak	Septicaemia and intestinal disorder	*S.* Typhimurium
6	2019	March	Outbreak	Intestinal disorder	*S.* Typhimurium
7	2015	March	Outbreak	Intestinal disorder	*S.* Typhimurium
7	2015	March	Outbreak	Intestinal disorder	*S.* Typhimurium
7	2017	July	Outbreak	Septicaemia and intestinal disorder	*S.* Typhimurium
7	2018	March	Outbreak	Intestinal disorder	*S.* Typhimurium
7	2019	April	Outbreak	Intestinal disorder	*S.* Typhimurium
7	2019	April	Outbreak	Intestinal disorder	*S.* Typhimurium
8	2015	July	Outbreak	Intestinal disorder	*S.* Typhimurium
8	2015	July	Outbreak	Intestinal disorder	*S.* Typhimurium
8	2016	March	Outbreak	Intestinal disorder	*S.* Typhimurium
8	2017	December	Outbreak	Septicaemia	*S.* Typhimurium
8	2018	May	Outbreak	Intestinal disorder	*Salmonella* serogroup B
9	2013	January	Outbreak ##	Intestinal disorder	*Salmonella* serogroup B
9	2017	March	Outbreak	Intestinal disorder	*S.* Typhimurium
9	2017	December	Outbreak	Intestinal disorder	*S.* Typhimurium
9	2019	March	Outbreak	Intestinal disorder	*S.* Typhimurium
10	2013	January	Outbreak ##	Intestinal disorder	*Salmonella* serogroup B
10	2013	January	Outbreak ##	Intestinal disorder	*Salmonella* serogroup B
10	2017	July	Outbreak	Septicaemia and intestinal disorder	*S.* Typhimurium
10	2017	December	Outbreak	Intestinal disorder	*S.* Typhimurium
11	2016	May	Outbreak	Intestinal disorder	*S.* Typhimurium
11	2017	July	Outbreak	Septicaemia and intestinal disorder	*S.* Typhimurium
12	2018	March	Outbreak	Intestinal disorder	*S.* Typhimurium
14	2018	February	Outbreak	Intestinal disorder	*S.* Typhimurium
14	2018	February	Outbreak	Intestinal disorder	*S.* Typhimurium
20	2016	April	Outbreak ##	Intestinal disorder	*S.* Typhimurium
22	2017	May	Outbreak ###	Intestinal disorder	*S.* Typhimurium
26	2016	June	Outbreak ##	Intestinal disorder	*S.* Typhimurium
217	2015	December	Outbreak	Intestinal disorder	*S.* Typhimurium
600	2013	October	Individual	Intestinal disorder	*S.* Dublin
2038	2017	December	Outbreak	Intestinal disorder	*S.* Enteritidis

* Outbreak defined as more than one animal with comparable clinical features at submission. ** ‘Age’ indicates the age of the submitted animal at death or abortion. # Hobby farm; ## rearing farm; ### buck fattening farm; the others are dairy goat farms.

**Table 4:** Antimicrobial susceptibility test results of all 42 isolates of *Salmonella* spp. from goats.

Antibiotics	AMEG Category ª	Number of Samples	Number (%) of Intermediate Isolates	Number (%) of Resistant Isolates
Amoxicillin-clavulanic acid	D	38	15 (39)	0 (0)
Ampicillin	D	42	0 (0)	40 (95)
Apramycin	C	38	0 (0)	0 (0)
4th generation cephalosporins	B	38	0 (0)	0 (0)
Cefotaxime	B	38	0 (0)	0 (0)
Colistin	B	42	1 (2)	0 (0)
Enrofloxacin	B	42	0 (0)	0 (0)
Florfenicol	C	38	24 (63)	12 (32)
Flumequine	B	38	2 (5)	0 (0)
Gentamicin	C	42	0 (0)	9 (21)
Neomycin	C	42	5 (12)	19 (45)
Spectinomycin	D	42	23 (55)	18 (43)
Sulfonamides	D	38	0 (0)	36 (95)
Tetracycline	D	38	0 (0)	35 (92)
Tiamulin *	C	38	0 (0)	38 (100)
Tilmicosin *	C	38	0 (0)	38 (100)
Streptomycin	C	38	7 (18)	23 (61)
Trimethoprim	D	38	0 (0)	27 (71)
Trimethoprim−sulfonamides	D	42	0 (0)	27 (64)
Tylosin *	C	38	0 (0)	38 (100)

ª EMA’s Antimicrobial Advice Ad Hoc Expert Group (AMEG) categorisation: B = Restrict; C = Caution; D = Prudent [20]. * The organism is intrinsically resistant to the indicated antimicrobial agent(s) [24].

**Table 5:** Summary of investigations on (dairy) goat farms with an outbreak of salmonellosis.

	Farm 1	Farm 2	Farm 3	Farm 4	Farm 5
Type farm	Dairy goat	Buck fattening	Dairy goat	Dairy goat	Dairy goat
Year outbreak	2017	2017	2015; 2016; 2017; 2018; 2019	2016; 2017; 2019	2018
Age of affected kids (days) #	0–7	22	5–10	6–10	5–12
Morbidity rate (%) #	Increased	50	Increased	30	Increased
Case fatality rate (%)	Unknown	100	50–70	25	Unknown
Serovar or serogroup	*S.* Typhimurium	*S.* Typhimurium	*S.* Typhimurium	*S.* Typhimurium	*S.* Typhimurium
MLVA profile	03-15-05-00-311	03-13-09-00-211	03−15−05−00−311(2016); 03-18-05-00-311 (2018) Other locations of farm 3 of the same owner: (A) Rearing 03−13−10−00−211 (2016). (B) Dairy goat 03−15−05−00−311 (2016).	03−13−09−00−211 (2017); 03-13-10-00-211 (2017); 03-13-11-00-211 (2019)	03-13-11-00-211 (2018)
Closed herd #	No	No	No	No	Unknown
Human disease #	Yes	Yes	Yes	No	No

# Based on anamnesis from farmer and/or veterinary practitioner.

## Data Availability

The data presented in this study are available on reasonable request from the corresponding author. The data are not publicly available due to privacy-sensitive information.
